# Effects of RNA Interference-Mediated Silencing of the Insulin-Like Androgenic Gland Hormone Gene on Growth and Gonad Development in the Swimming Crab (*Portunus trituberculatus*)

**DOI:** 10.3390/ani16091413

**Published:** 2026-05-05

**Authors:** Weiren Zhang, Ronghua Li, Chuan He, Changkao Mu, Chunlin Wang, Ce Shi, Weiwei Song

**Affiliations:** 1 Key Laboratory of Aquacultral Biotechnology (Ningbo University), Chinese Ministry of Education, Ningbo 315211, China; 2 Collaborative Innovation Center for Zhejiang Marine High-Efficiency and Healthy Aquaculture, Ningbo 315211, China; 3 Key Laboratory of Green Mariculture (Co-Construction by Ministry and Province), Ministry of Agriculture and Rural, Ningbo 315211, China

**Keywords:** IAG, *Portunus trituberculatus*, knockdown, growth, sex reversal

## Abstract

The insulin-like androgenic gland (IAG) gene is considered a crucial factor in the process of sexual differentiation in decapod crustaceans. In this study, we characterized the structural features and ontogenetic expression profile of the *IAG* gene in the swimming crab *P. trituberculatus* (*Pt-IAG*). To further elucidate its biological roles, we performed long-term RNA interference (RNAi) to evaluate the physiological impacts of sustained *Pt-IAG* suppression. Results showed that the gene structure of *Pt-IAG* is conserved with the characteristic B-C-A polypeptide arrangement that is representative of decapod *IAG*s. Transcripts of *Pt-IAG* were detectable as early as the zygote stage, which may imply its potential role during early-stage development. Although no completesex reversal was observed, continuous knockdown of *Pt-IAG* resulted in a decrease in body weight and a delay in gonad development in both sexes, underscoring its pleiotropic role in coordinating growth and maturation. Furthermore, the silencing of *Pt-IAG* led to a significant downregulation of insulin-like growth factor-binding protein (*Pt-IGFBP*), and a decrease in insulin-receptor (*Pt-IR*) expression was observed in males. Notably, the *Pt-IR* gene was not detected in female ovaries. These findings suggest that IAG may modulate male development through an insulin-like signaling axis; however, the absence of ovarian *Pt-IR* expression implies the potential existence of alternative regulatory pathways in females. Collectively, this study identifies a dual regulatory role of IAG in both growth and reproduction, providing a specific theoretical basis for developing sex-control and growth-optimization strategies in crab aquaculture.

## Simple Summary

The insulin-like androgenic gland (IAG)hormone controls sexual development in decapod crustaceans, including crabs and shrimp. In some species, knocking down this gene can transform males into females—a technique highly valued in aquaculture because female crabs often command higher market prices. This study characterized the gene structure and biological roles of this hormone in the swimming crab (*P. trituberculatus*), and further examined whether suppressing it would trigger sex reversal. Our results showed that the cDNA sequence of *Pt-IAG* is highly similar to those in other decapods. While reducing the hormone’s activity slowed growth and delayed the development of reproductive organs in both males and females, this treatment was not sufficient to induce sex reversal. These findings suggest that the hormone influences overall growth and development, rather than acting solely as a switch for sex determination. Understanding these differences helps scientists develop better approaches for improving crab farming and may eventually lead to methods for producing monosex populations.

## 1. Introduction

IAG is a pivotal endocrine factor in male crustaceans, serving as the driver of sexual differentiation and gonadal development [1,2]. While the manipulation of *IAG* has successfully induced complete sex reversal in certain caridean shrimp, such as *Macrobrachium rosenbergii* [3,4] and *Exopalaemon carinicauda* [5], similar interventions have failed to achieve full phenotypic transition in other crustaceans. This discrepancy suggests that the functions and regulatory mechanisms of IAG may exhibit significant evolutionary divergence across different decapod lineages [6,7]. In the swimming crab *Portunus trituberculatus*, *Pt-IAG* has been identified and its tissue distribution documented [8]. Previous transcriptomic analyses by our group revealed high expression of *Pt-IAG* during the megalopae stage, indicating its involvement in early ontogenetic development [9]. Furthermore, the recent detection of *Pt-IAG* transcripts in both sexes [10] challenges the classical “male-specific” paradigm and hints at a pleiotropic role in growth and reproduction. Despite these advances, critical research gaps persist. Notably, functional in vivo validation of *Pt-IAG* remains inadequate, and its physiological role in females is poorly defined. Furthermore, the majority of functional studies have relied on short-term RNA interference (RNAi), which fails to recapitulate the sustained physiological impacts of IAG across extended developmental periods.

The IAG peptide belongs to the insulin-like peptide (ILP) superfamily and is hypothesized to signal through a conserved pathway involving binding proteins and transmembrane receptors [11]. In *Cherax quadricarinatus*, an IGFBP homolog was confirmed to bind specifically to IAG [12]. Similarly, silencing of *IGFBP* in *M. rosenbergii* and *Macrobrachium nipponense* significantly suppressed *IAG* transcription, suggesting a robust regulatory feedback loop [11,13]. Moreover, IAG is transported by IGFBP within the hemolymph [14], emphasizing their close functional interdependence. The insulin receptor (IR), a member of the receptor tyrosine kinase subfamily, is recognized as the primary mediator of these ILP signals [15]. Evidence from *Sagmariasus verreauxi* shows that IAG activates the MAPK/ERK pathway via IR [16], while co-localization of *Mr-IR* and *Mr-IAG* further validates this receptor–ligand pairing [15]. In *P. trituberculatus*, previous studies have established the co-localization and feedback regulation of *Pt-IGFBP* and *Pt-IAG*, which also constitute a functional signaling axis that is recognized and responded to by the receptor *Pt-IR* [8,17,18]. Thus, analysis of these pathway-related genes can clarify the mechanisms of IAG-mediated signaling, investigate the response of downstream target engagement, and identify physiological changes invoked under chronic IAG inhibition.

RNAi is considered an effective method to study the function of targeted genes [19,20]. Although traditionally mediated by long dsRNA or shRNA—which are intracellularly processed by Dicer into siRNAs—this approach often encounters challenges in crustaceans, including delayed onset and potential off-target-induced immune responses due to the generation of heterogeneous siRNA pools [21]. In contrast, chemically synthesized siRNA systems offer advantages such as rapid onset and well-defined metabolic cycles, enabling precise control over dosage and concentration with high reproducibility [21]. The efficacy of siRNA-mediated silencing has been validated in characterizing IAG functions in *M. rosenbergii* [15] and *Eriocheir sinensis* [22], as well as *Sox* genes in *P. trituberculatus* [23], providing a robust toolkit for exploring crustacean sexual development. These advancements provide a foundation for potentially manipulating sexual development in economically significant crustaceans.

The swimming crab *P. trituberculatus* is an economically important crab in Southeast Asia, with capture fisheries in China alone reaching 97,700 tons in 2024 [24]. Given the high market value of sexually mature females, which are prized for ther nutrient-rich gonads, establishing all-female monosex culture has become a strategic priority for the industry [25]. Although IAG is traditionally defined as a male factor, its expression in the ovaries and hepatopancreas of *Scylla paramamosain* and *Callinectes sapidus* suggests a potential role in female reproductive regulation [26,27]. Establishing a clear link between IAG signaling and gonadogenesis is therefore not only of fundamental biological interest but also of significant practical value for sex-control breeding technologies. To address these challenges, the present study was designed with three primary objectives: first, to characterize the ontogenetic expression profiles of *Pt-IAG* during early life stages, from the zygote to larval stages; second, to evaluate the physiological impacts of sustained gene silencing via long-term RNAi on growth performance and gonadal morphogenesis; and third, to investigate the underlying regulatory signaling by monitoring the expression responses of two key components: the binding protein *Pt-IGFBP* and the receptor *Pt-IR.*Collectively, these investigations aim to provide a theoretical foundation for understanding the pleiotropic mechanisms of IAG in regulating growth and reproduction, ultimately supporting the development of efficient all-female monosex culture models.

## 2. Materials and Methods

### 2.1. Overview of Experimental Design

**Figure 1:**
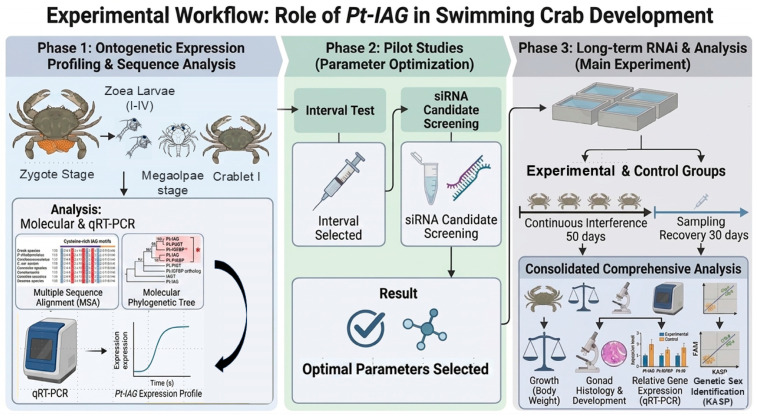
The schematic framework of the experimental main workflow.

To systematically investigate the role of *Pt-IAG*, the main experimental workflow was structured into three sequential and interdependent phases (Figure 1). (1) Ontogenetic expression profiling, where samples were collected from zygotes to juvenile stage I to establish the ontogenetic expression profile of *Pt-IAG*; (2) pilot studies, conducted on juvenile crabs to optimize siRNA sequences, dosages, and injection intervals; and (3) long-term RNAi experiment, utilizing the optimized parameters from the pilot studies to evaluate the physiological and molecular impacts of sustained *Pt-IAG* suppression over a 50-day period followed by a 30-day recovery phase.

### 2.2. Sample Collection

All the larvae and crablets used in the experiment were collected from the Choupijiang Aquatic Products Limited Company (Ningbo, Zhejiang, China). All aquaculture experiments in this study were conducted under the following conditions: natural photoperiod, with temperature and salinity constant at 22 ± 1 °C and 25 ± 1 ppt; water was exchanged 50% every 3–4 days; quality parameters of the water (pH 7.0–9.0, dissolved oxygen > 4 mg/L, ammonia nitrogen < 0.8 mg/L, and nitrite < 0.15 mg/L) were maintained throughout the study following Wu et al.’s (2010) method [28].

Female crabs (*n* = 10, weight 250–300 g) were individually housed in white plastic buckets with a volume of 50 L and fed live manila clam (*Ruditapes philippinarum*) twice daily (3–5% of body weight) until hatching. Twenty days later, five berried crabs were randomly chosen, and eggs in the zygote, cleavage, blastula, eye-pigment and heartbeat stages were collected from the abdomen of female crabs. After hatching, the larvae of each female were transferred to separate concrete tanks (5 × 5 × 1 m^3^) and fed with brine shrimp (*Artemia*) at 2–3% of their body weight, while juvenile crabs were fed live *R. philippinarum* at 3–5% of their body weight, following the protocol of Geng et al. (2025) [29]. For the larval development stages, the larvae from five female swimming crabs were collected at the zoea I, zoea II, zoea III, zoea IV, megalopae and juvenile crab I stages. The sampling strategy for each developmental stage primarily followed Wang et al. (2020) [30]. To ensure sufficient biomass and representative genetic diversity, a stage-specific pooling strategy was adopted for biological replicates (*n* = 6): for zygote to megalopae stages, each biological replicate consisted of a pool of 10–20 individuals. For the juvenile crab I (C I) stage, each biological replicate consisted of a pool of 6 individuals. Each developmental stage was confirmed by light microscopy with reference to the established criteria (Table S1) [31,32,33]. The samples were frozen immediately in liquid nitrogen and stored at −80 °C for subsequent RNA isolation and gene expression analysis.

Juveniles at the crab I stage were collected from five female crabs, with 400 individuals randomly collected from each female, ensuring a rearing density of 200 individuals/m^2^ [34]. The collected juveniles of crab I stage were pooled and transferred to another cement pond (2 × 5 × 1 m^3^), and they were reared until the third juvenile crab stage (C III) for the RNAi experiment. During the RNAi experiment, the same water environmental conditions and feeding strategy were maintained as described above. To ensure random sampling of individuals for the experiment, the rearing pond was evenly divided into several equally sized sampling zones, and an equal number of individuals were collected from each zone, thereby achieving randomization.

### 2.3. The siRNA-PtIAGPreparation

The cDNA sequence of the *Pt-IAG* gene (GenBank accession no. MH119940.1) was downloaded and analyzed by Expasy Translate (http://web.expasy.org/translate/ (accessed on 6 December 2025)) to predict its amino acid sequence. To confirm the isoform identity of *Pt-IAG*, its sequence was aligned with several confirmed *IAG* gene sequences (Table 1), as well as with insulin, gonadulin, and relaxin sequences described by Veenstra (2020) [35]. Multiple sequence alignment and the construction of a neighbor-joining phylogenetic tree were performed using Clustal Omega (v1.2.4) [36] and MEGA (v11) [37], respectively, with default parameters. Four pairs of siRNA-*PtIAG* targeting different sites of the cDNA sequence were designed (Table 2) using the Designer of Small Interfering RNA (DSIR) webtool [38] and subsequently synthesized by GenePharma (Shanghai, China). To further ensure specificity, we performed an off-target analysis of the siRNA guide strand sequences using NCBI BLASTn (https://blast.ncbi.nlm.nih.gov/Blast.cgi (accessed on 6 December 2025)) following the method described by Li et al. (2025) [39]. The parameters were set as follows: the database was set to “nt”, organism was restricted to *P. trituberculatus*, and the program selected was “Somewhat similar sequences (blastn)”. The results showed that, apart from the target gene, the designed siRNA sequences did not exhibit matches to any other transcripts of *P. trituberculatus*, indicating a low risk of off-target effects.

### 2.4. Injection Interval, siRNA-PtIAG and Dosage Optimization

Crablets (weight 2.12 ± 1.23 g, at VI stage) were used for the determination of optimal injection interval in the pilot study. All the crablets in this experiment were individually housed in a separate culture basket to prevent cannibalism. After a 3-day acclimation period, a pilot study was conducted to determine the optimal injection interval. Sixty healthy male crablets were randomly allocated into four treatment groups (each with three replicates): one control group (no injection) and three experimental groups. Crabs in the experimental groups were injected at the base of the pleopods with physiological saline solution (1 μL/g body weight). The saline composition was (in mM): 440 NaCl, 11 KCl, 13.3 CaCl_2_, 26 MgCl_2_, 26 Na_2_SO_4_, and 10 HEPES, pH 7.4 with NaOH [42]. Injection intervals were set as 1, 2, and 5 days for the three experimental groups. The experiment lasted for 10 days. Mortality was recorded daily.

To test the effect of different types of siRNAs, 650 healthy male crabs at stage III (body weight 0.5 ± 0.1 g) were randomly selected to perform a pilot study. They were divided into thirteen groups with 50 crabs in each group, including one control group and twelve experimental groups. The control group was injected with 1 μL/g body weight of physiological saline solution at the base of the pleopods. Each experimental group was assigned a single siRNA type with a specific dosage (0.5, 1, or 2 μg/g body weight) [15], and each crab was injected only once to evaluate the short-term silencing efficiency during this screening phase. Experimental groups were named based on the target primer sequence and injected dosage, formatted as “[Primer Name]-[Dosage in μg/g].” The groups included: Pt-104-0.5, Pt-104-1, Pt-104-2, Pt-301-0.5, Pt-301-1, Pt-301-2, Pt-598-0.5, Pt-598-1, Pt-598-2, Pt-744-0.5, Pt-744-1, and Pt-744-2. At 0, 24, 48, 72, 96, and 120 h post-injection, six individuals were randomly selected from each group [8]. They were anesthetized by immersion in an ice–water slurry (0–2 °C) for 10–15 min until motionless. Subsequently, the androgenic gland was dissected, immediately snap-frozen in liquid nitrogen, and stored at −80 °C for subsequent RNA isolation.

### 2.5. RNAi

Based on the pilot study, siRNA-Pt-598 was selected for the formal experiment due to its superior knockdown efficiency compared to other candidates. At a dosage of 1 μg/g, siRNA-Pt-598 achieved the maximum reduction in *Pt-IAG* mRNA expression. Consequently, a 5-day injection interval was adopted for the long-term study to ensure sustained gene silencing while minimizing handling stress. A total of 400 individuals (male:female 1:1) of crablet III (body weight 0.5 ± 0.1 g) were randomly divided into two groups (siRNA-PtIAG experimental group and control group) with four replications (concrete tanks of 5 × 5 × 1 m^3^). To minimize handling stress and mortality, each crab was injected every five days over the 50-day period. While repeated injections may introduce cumulative handling stress, we mitigated this risk by using a consistent physiological saline control group to isolate the specific effects of *Pt-IAG* silencing. Furthermore, the 5-day interval was chosen based on the pilot study to balance gene silencing efficiency with the physiological recovery of the crabs. On the 50th day, 24 h after the final injection, the body weight of twelve individuals of each group (male:female 1:1) was recorded. Thirty-six crabs (male:female 1:1) were randomly sampled from each group for sample collection. Prior to sampling, crabs were euthanized in ice–water slurry (0–2 °C) for 10–15 min until motionless. For histological analysis, gonads were dissected and fixed in 4% paraformaldehyde. For molecular analysis, gonads and androgenic glands, as well as chela muscle tissue, were dissected, immediately snap-frozen in liquid nitrogen, and stored at −80 °C for subsequent RNA and DNA extraction, respectively. To ensure the objectivity of the molecular analysis, six biological replicates were randomly selected from each group using a computer-generated random sequence. This standardized sampling approach was adopted to minimize experimental bias and ensure that the analyzed individuals were representative of the overall population treatment effect.

Following this, all remaining crabs were cultured for an additional 30 days without any injection, while maintaining the same aquaculture protocols. On the 80th day, the body weight of twelve individuals of each group (male:female 1:1) was recorded. Gonads and androgenic glands, as well as chela muscle tissue of 18 samples of each group (male:female 1:1), were dissected, immediately snap-frozen in liquid nitrogen, and stored at −80 °C for subsequent RNA and DNA extraction, respectively.

### 2.6. Quantitative Real-Time PCR (qRT-PCR)

Relative mRNA expression levels of *Pt-IAG*, *Pt-IGFBP* and *Pt-IR* at different development stages were assessed by qRT-PCR. Total RNA from the different development stages, muscle, testis, ovary and androgenic glands were isolated using TRIzol reagent (Ambion, Inc., Austin, TX, USA) according to the manufacturer’s instructions. The integrity of RNA was tested via gel electrophoresis, whileconcentration and purity (A260/A280 and A260/A230 ratios) were measured using a NanoDrop 2000 spectrophotometer (Thermo Scientific, Waltham, MA, USA). The RNA samples with A260:A280 values between 1.9 and 2.1 and A260:A230 values higher than 2.0 were taken for further experiments. First-strand cDNA was synthesized from 1 µg of total RNA using the HiFi-Script cDNA Synthesis Kit with gDNA removal (CWBIO, Beijing, China).

Primers used in this study are listed in Table 2. The *Pt-IAG* primer set was designed using Primer6 software, while those for *Pt-IGFBP* and *Pt-IR* were adopted from Jiang et al. (2020) [8]. Ribosomal protein L18 (RPL-18) served as the reference gene [43]. For each primer pair, PCR reaction efficiency was computed using LinRegPCR (v20210614) [44] from the non-baseline corrected amplification data with 6 replicates (Table S2) [45]. The efficiencies ranged from 90% to 101%. The qRT-PCR analysis was performed using TB Green^®^ Premix Ex Taq II (TaKaRa Bio Inc., Dalian, China). The reaction system (20 μL) consisted of 10 μL of TB Green Premix Ex Taq II, 0.4 μM primers and 2 μL of cDNA template. The qRT-PCR was performed on a LightCycler 480 real-time PCR instrument (Roche Diagnostics, Ltd., Burgess Hill, UK) with the following program: pre-denaturation at 95 °C for 5 min, followed by 40 cycles at 95 °C for 10 s and 60 °C for 15 s. and a final curve analysis of 1 cycle at 95 °C for 15 s, 60 °C for 1 min and 95 °C for 15 s. Three technical replicates were performed for each qRT-PCR reaction of each sample. Relative gene expression was calculated using the 2^−ΔΔCT^ method [46]. To evaluate the effectiveness of the RNAi, qRT-PCR validation was performed to assess knockdown efficiency, defined as the percentage reduction in target gene expression in the siRNA-treated group compared to the control group [47].

To ensure compliance with the MIQE guidelines and avoid the use of unstable reference genes, the specificity of the primer pairs was verified by the appearance of a single peak in the dissociation curve analysis with 18 replicates (Figure S1). Furthermore, the expression stability of the reference gene (RPL-18) was statistically validated. An additional three mature crabs were collected, and four tissues (muscle, testis, androgenic gland, and ovary) were dissected. The expression level of *RPL-18* was quantified by qRT-PCR, with three technical replicates per sample. The Ct values from each tissue were analyzed using BestKeeper (v1) to assess expression stability [48]. The results showed that *RPL-18* exhibited a standard deviation (SD) of 0.63, a coefficient of variation (CV) of 3.31%, and a BestKeeper score of 0.49, which was below the recommended thresholds (SD < 1.0), indicating highly stable expression across different tissues and confirming its suitability as a reference gene for normalization (Table S3).

### 2.7. Histological Analysis of Gonads

Gonads were fixed in 4% paraformaldehyde for histological analysis. Following fixation, samples were dehydrated in a graded ethanol series, cleared with xylene, infiltrated with paraffin at 60 °C, embedded, and sectioned at 5 μm. Sections were stained with hematoxylin–eosin and examined under a light microscope. Images were captured and analyzed using the integrated ScopeImage 9.0 software. For each gonad sample, tissues were serially embedded, and every fifth section was selected to obtain three non-consecutive sections per sample, ensuring representative sampling and avoiding repeated counts from consecutive sections. For each gonad sample, at least three histological sections were examined. From each section, 4–5 fields of view were randomly selected for analysis to identify cell types and measure relevant cellular diameters. All histological analyses were performed in a blinded manner, with histological scoring conducted independently by two observers. Ovarian developmental stages were classified according to the microscopic assessment criteria established for *P. trituberculatus* by Che et al. (2018) [49] and Feng et al. (2023) [50], as summarized in Table S4. Similarly, testis stages were determined following the microscopic evaluation criteria described by Che et al. (2019) [51] and summarized in Table S5. Finally, a quantitative morphometric analysis was performed using ScopeImage 9.0 software to calculate specific indices for various cell types within each histological section, enabling an objective assessment of individual developmental status. The developmental status of the gonads was quantified using the Gonadal Coverage Area (GCA), calculated as follows [52]:

$$GCA\;(\%)=\frac{A_{gonad}}{A_{total}}\times 100$$

where $A_{gonad}$ represents the area occupied by a specific germ cell type or tissue component, and $A_{total}$ represents the total area of the histological section image. These indices quantify the proportion of specific germ cell types, where a higher area percentage of mature cells (e.g., spermatozoa or vitellogenic oocytes) serves as a quantitative marker for advanced gonadal maturation, allowing for objective comparison of developmental progression between groups. The staging frequency was defined as the ratio of the number of individuals at a specific developmental stage to the total number of sampled individuals.

### 2.8. Identification of Genetic Sex

In this study, the sex of crabs was determined using two strategies: phenotypic sex identification and genetic sex identification. For phenotypic sex identification, sex was determined based on abdominal morphology and gonadal histology. Female crabs were identified by a nearly round abdominal flap and the presence of ovarian tissue, while males were identified by a triangular abdominal flap and the presence of testicular tissue. To confirm genetic sex, the Kompetitive Allele-Specific PCR (KASP) method targeting the locus Ptr67655 was performed following Lu et al. (2021) [40]. Genomic DNA was extracted from muscle samples of thirty-six muscle samples at 50 days and eighteen muscle samples at 80 days (male:female 1:1) using the EZNA^®^ Tissue DNA Kit (Omega, Inc., Norcross, GA, USA). The KASP reaction was carried out in a 10 μL volume on a LightCycler480 instrument (Roche Diagnostics Ltd., Burgess Hill, UK) with primers and probes listed in Table 2.

### 2.9. Statistical Analysis

For the long-term RNAi experiment and subsequent physiological analyses, the individual crab was defined as the primary unit of biological replication. To ensure statistical independence, individuals were housed in separate physical compartments within the tanks to prevent social interaction or cannibalism. The culture tanks served as parallel environmental units to maintain experimental consistency. All statistical analyses were performed using SPSS 27.0 software (IBM Analytics, Richmond, VA, USA). The normality of data distribution was verified using the Shapiro–Wilk test, and homogeneity of variances was assessed using Levene’s test before applying parametric tests. For comparisons between two groups, an independent-samples *t*-test was applied to analyze quantitative morphometric data, body weight, and the expression changes in *Pt-IAG*, *Pt-IGFBP* and *Pt-IR*. Additionally, Cohen’s d was calculated to evaluate the effect size for key parameters, ensuring that the observed differences were both statistically significant and biologically meaningful (Table S6). For comparisons among multiple groups, one-way analysis of variance (ANOVA) was performed, followed by Tukey’s posthoc test, which was used for the expression of *Pt-IAG* at different developmental stages, survival rates under different injection intervals, and the expression changes in *Pt-IAG* during optimal siRNA screening. Statistical significance was set at *p* < 0.05 and *p* < 0.01, denoting significant and highly significant differences, respectively, and significance is denoted with asterisks or different letters in the figures.

## 3. Results

### 3.1. The Protein Sequence Prediction of the Pt-IAG Gene

**Figure 2:**
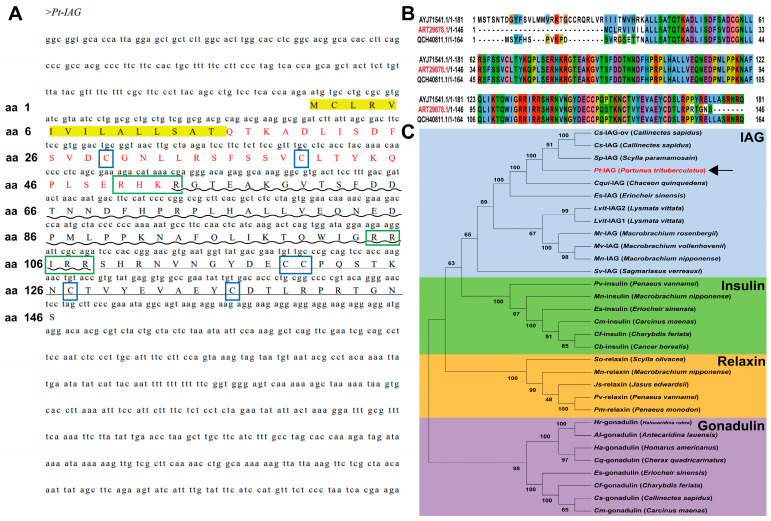
Sequence and phylogenetic analysis of *Pt*-*IAG*. Note: (**A**) Schematic representation of the deduced *Pt*-*IAG* precursor protein structure. The yellow color indicates signal peptide, red font indicates B chain, ~ indicates C peptide, ——indicates A-chain, blue box indicates Cys residue, and green box indicates the cutting site of Arg C protease. (**B**) Multiple sequence alignment of *Pt*-*IAG* with homologous sequences (GenBank accession no. QCH40811.1 and AYJ71541.1). (**C**) Neighbor-joining phylogenetic tree of insulin and related peptide amino acid sequences from decapods.

The validated cDNA sequence of *Pt-IAG* and its deduced amino acid (aa) sequence are shown in Figure 2A. The open reading frame encodes a 146-amino acid prepropeptide with a predicted signal peptide (aa 1–16), B-chain (aa 17–52), C-peptide (aa 53–108), and A-chain (aa 109–146), consistent with the typical organization of ILPs. Conserved features include the dibasic cleavage sites (RRIRR and RHKR) and three conserved disulfide bonds (Cys29–Cys120, Cys40–Cys136, and Cys119–Cys127).

Two additional amino acid sequences of IAG (GenBank accession no. QCH40811.1; AYJ71541.1) were also downloaded from the NCBI database. Multiple sequence alignment (Figure 2B) revealed differences in certain amino acid residues within the signal peptide and C-terminus among these sequences. To examine the evolutionary relationship of *Pt-IAG* with other *ILPs*, we conducted a phylogenetic analysis using IAG, insulin, gonadulin, and relaxin sequences (Figure 2C). The resulting tree resolved four distinct clades corresponding to these subfamilies, with *Pt-IAG* clustering within the IAG clade.

### 3.2. Expression Profiles of Pt-IAG During Zygote–Larval Development

**Figure 3:**
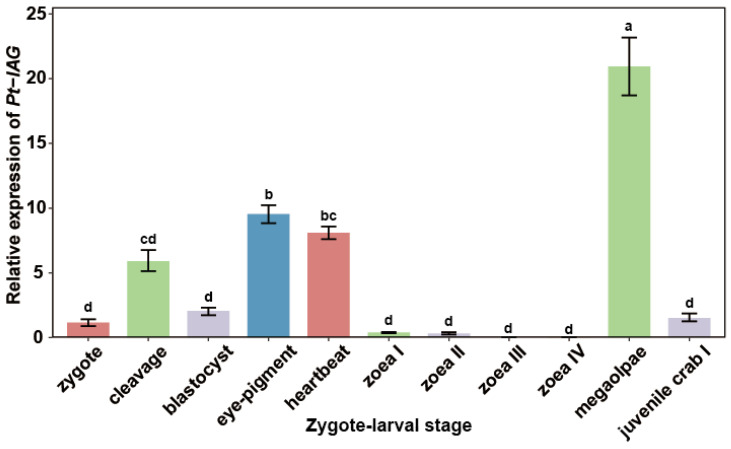
Relative expression of the *Pt-IAG* gene during zygote–larval development. Note: Data are presented as mean ± SD (*n* = 6, three technical replicates were performed for each sample) and were normalized to the zygote group. Significant differences among groups are indicated by different letter labels (one-way ANOVA, followed by post hoc Tukey’s multiple-group comparison, *p* < 0.05.

To ensure the reliability of developmental staging, we referenced the fecundity range of *P. trituberculatus* (140.64 ± 40.80 × 10^4^ eggs per female) [28]. Throughout the rearing process, over 90% of individuals at each developmental stage were confirmed to be healthy under microscopy. The expression level of the *Pt-IAG* gene across different developmental stages was determined by qRT-PCR. Results revealed that *Pt*-*IAG* was expressed during the zygote, cleavage, eye-pigment, and megalopae stages. A significant upregulation occurred as the embryo transitioned from the blastocyst to the eye-pigment stage, with a 4.6-fold increase in expression (*p* = 2.7 × 10^−8^). After hatching, expression remained consistently low throughout the entire zoea stages (I–IV), at only 1.89–33.5% of the zygote stage level. Asecond sharp increase was observed during the metamorphosis from zoea IV to the megalopae stage, with a 956.7-fold increase in expression (*p* = 4.4 × 10^−19^). Finally, expression dropped significantly again upon reaching the juvenile crab I stage, reaching levels similar to those of the zygote stage (Figure 3).

### 3.3. Optimal Injection Interval, siRNA-PtIAG and Dose

**Figure 4:**
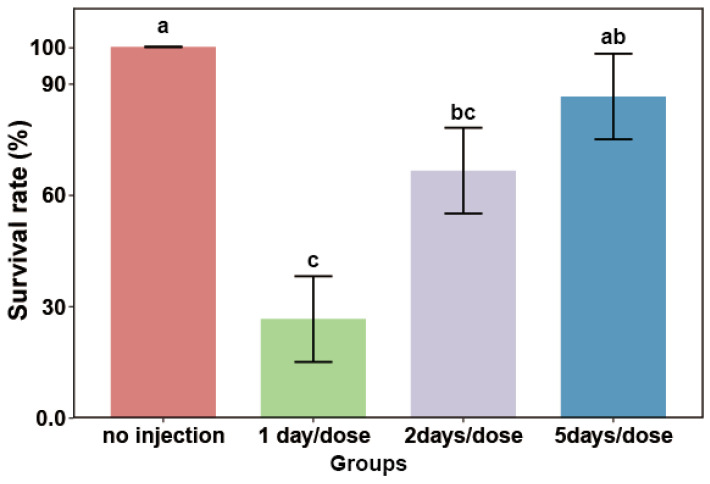
Survival rate of the crablet at different injection intervals. Note: Significant differences among groups are indicated by different letter labels (one-way ANOVA, followed by post hoc Tukey’s multiple-group comparison, *p* < 0.05).

In order to decrease the mortality of crabs due to long-term injections, the present study compared the survival rate at different injection intervals. The results showed that the survival rate gradually increased with the prolongation of the injection interval for crabs. Specifically, the 1-day/dose group exhibited a significantly lower survival rate (26.67%) compared to both the no-injection control (*p* = 0.026) and the 5-day/dose group (86.67%; *p* = 0.039) after ten days of treatment. Although the survival rate of the 2-day/dose group was higher (66.67%), it did not differ significantly from the 1-day/dose group (*p* = 0.100). Notably, the survival rate in the 5-day/dose group remained comparable to the control (Figure 4).

**Figure 5:**
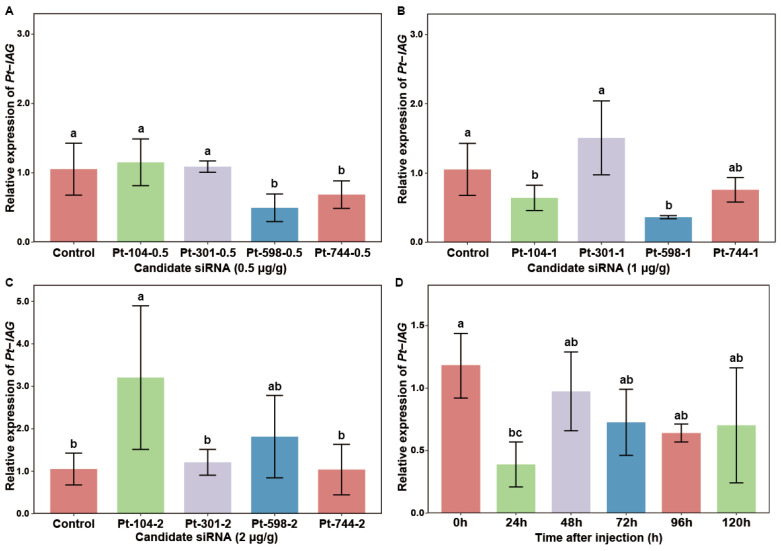
Relative expression of the *Pt-IAG* gene. Note: (**A**–**C**) The relative expressions of *Pt-IAG* 24 h post-injection at doses of (**A**) 0.5, (**B**) 1, and (**C**) 2 μg/g for each siRNA. Data are presented as mean ± SD (*n* = 6, three technical replicates were performed for each sample) and were normalized to the control group. (**D**) Arelative expression of *Pt-IAG* at different timepoints post-injection with siRNA-Pt-598. Data are presented as mean ± SD (*n* = 6, three technical replicates were performed for each sample) and were normalized to the 0 h group. Significant differences among groups are indicated by different letter labels (one-way ANOVA, followed by post hoc Tukey’s multiple-group comparison, *p* < 0.05).

To determine the optimal interference site and dosage, the silencing efficiency of four siRNA candidates was evaluated via qRT-PCR at 24 h post-injection. The results revealed distinct dosage-dependent inhibitory effects (Figure 5A–C). At a dosage of 0.5 μg/g, both siRNA-Pt-598 and siRNA-Pt-744 significantly suppressed *Pt-IAG* transcript levels (*p* < 0.05). siRNA-Pt-598 achieved a 2.12-fold reduction with a knockdown efficiency of 53.0% (CV = 40.20%, *p* = 0.009), while siRNA-Pt-744 demonstrated the highest efficiency in this group at 34.89% (CV = 28.91%, *p* = 0.047) (Figure 5A). At the 1 μg/g dosage, siRNA-Pt-104 and siRNA-Pt-598 emerged as the most effective candidates (Figure 5B). siRNA-Pt-104 reduced transcription by 1.63-fold (38.97% efficiency; *p* = 0.040). Notably, siRNA-Pt-598 exhibited a more robust inhibitory effect, resulting in a 2.89-fold decrease and a peak knockdown efficiency of 65.8% (*p* = 0.006, CV = 6.24%). In contrast, no significant gene silencing was observed among the groups treated with 2 μg/g siRNA (Figure 5C). Instead, a significant upregulation of *Pt-IAG* mRNA was detected in the siRNA-Pt-104 group (*p* = 0.046).

As the most significant knockdown effect of siRNA-Pt-598 with an injection dosage of 1 μg/g was observed at 24 h (knockdown efficiencies: 65.5%), with the lowest coefficient of variation (CV_1 μg/g_ = 6.24%) among all treatment groups, qRT-PCR analysis was performed for the relative expression of *Pt-IAG* gene at different times in this experimental group (Figure 5D). The interference effect of siRNA-Pt-598 on *Pt-IAG* mRNA levels exhibited a clear time-dependent recovery pattern. Following a single injection, *Pt-IAG* transcription reached its nadir at 24 h, showing a significant 3.04-fold decrease compared to the 0 h baseline (*p* = 0.022). By 48 h, the expression level had recovered to 82.25% of the initial value. Statistical analysis confirmed that from 48 h through 120 h, there were no significant differences in *Pt-IAG* transcript levels compared to the 0 h control (*p* > 0.05).

### 3.4. Changes in Body Weight and Gonadal Histology

**Figure 6:**
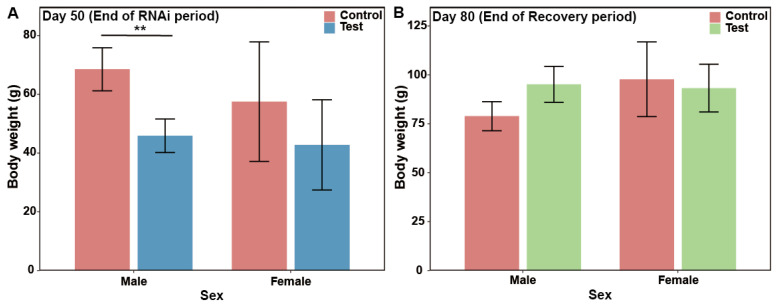
Body weight comparison of different groups. Note: (**A**,**B**) The body weightson the 50th day and the 80th day. ** indicates highly significant difference (*p* < 0.01). Significant differences between groups are indicated by asterisks based on independent-samples *t*-tests.

The growth of *P. trituberculatus* was significantly affected by *Pt-IAG* knockdown, followed by a notable compensatory growth phase after the cessation of RNAi (Figure 6A,B). On day 50 of continuous interference, the body weight (BW) of male crabs in the control group was 49.4% greater than that of those in the experimental group (BW_control_ = 68.54 ± 7.34 g vs. BW_experimental_ = 45.89 ± 5.73 g; *p* = 0.002) (Figure 6A). In contrast, no significant difference in BW was observed between the female crabs of the two groups at this stage. Following the cessation of RNAi (from day 50 to day 80), a rapid recovery in growth was observed in both sexes. By day 80, the initial growth gap had closed; no significant differences in BW were detected between the control and experimental groups for either males (BW_control_ = 78.89 ± 7.42 g vs. BW_experimental_ = 95.17 ± 9.21 g; *p* = 0.08) or females (BW_control_ = 97.74 ± 19.12 g vs. BW_experimental_ = 93.21 ± 12.22 g; *p* = 1.00) (Figure 6B).

**Figure 7:**
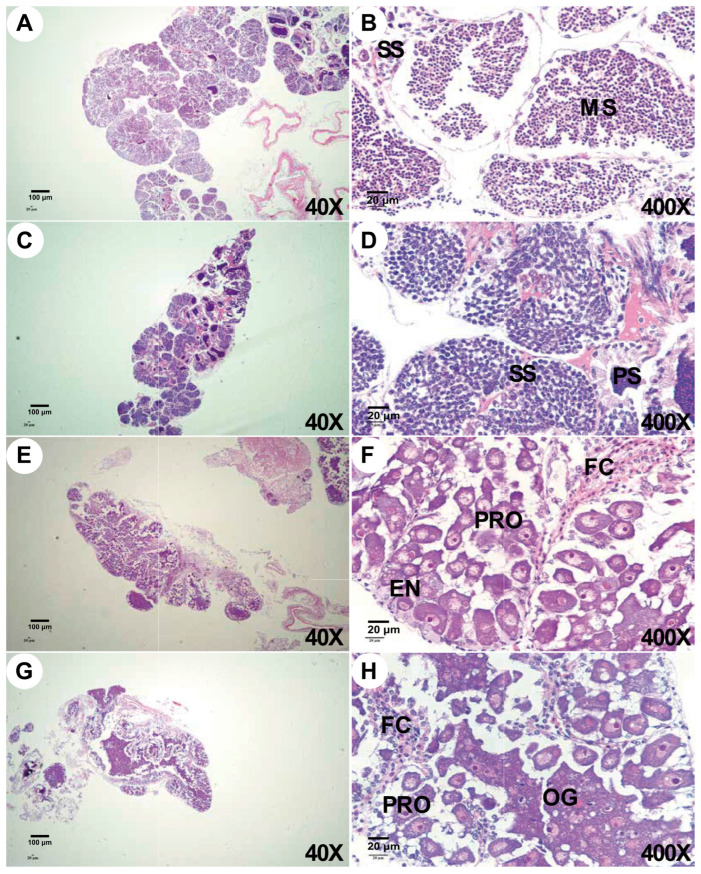
Microstructures of male and female gonads of the two groups. Note: (**A**–**D**) The male gonad of the control group and the experimental group, respectively. (**E**–**H**) The female gonad of the control group and the experimental group, respectively. MS: mature sperm;PS: primary spermatocyte; SS: secondary spermatocyte; FC: follicular cell, PRO: previtellogenic oocytes, EN: endogenous vitellogenic oocyte, OG: oocytes. Scale bars: 100 μm for (**A**,**C**,**E**,**G**) images; 20 μm for (**B**,**D**,**F**,**H**) images.

The histology of the gonads in both groups is presented in Figure 7. In the control groups (Figure 7A,B), the testes reached the spermatophore formation stage (staging frequency = 77.78%), characterized by a high density of mature spermatozoa (GCA = 75.73 ± 12.14%). Conversely, the experimental groups (Figure 7C,D) exhibited a marked developmental arrest, remaining predominantly at the secondary spermatocyte stage (staging frequency = 88.89%). The ratio of secondary spermatocytes was 83.64 ± 10.35%, while the percentage of mature spermatozoa was only 4.8 ± 1.47% in the experimental group, which was significantly decreased compared with the control group (*p* = 0.008).

The control ovaries (Figure 7E,F) progressed to early-stage-II (staging frequency = 88.89%), featuring ellipsoidal previtellogenic oocytes (PRO) (GCA = 74.93 ± 9.06%; mean length = 31.22 ± 0.31 μm) and the presence of endogenous vitellogenic oocytes (EN) (mean length up to 49.04 ± 0.21 μm) at the periphery. In the experimental groups (Figure 7G,H), ovarian development was delayed at the end of stage I (staging frequency = 66.67%). The ovaries were dominated by oogonia (OG) (GCA = 33.82 ± 2.25%) with a mean length (9.20 ± 0.15 μm). The overall ratio of previtellogenic oocytes was significantly lower (GCA = 21.6 ± 6.36%) compared to the control group (*p* = 0.002).

### 3.5. Genetic Sex Genotyping

**Figure 8:**
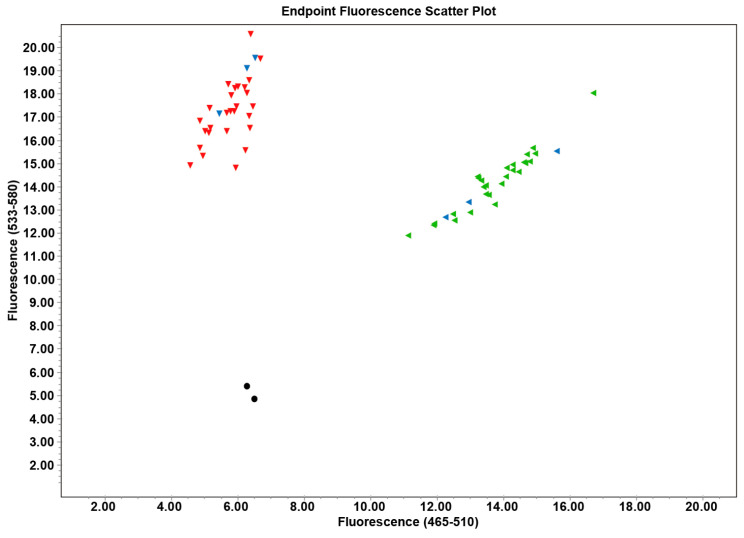
Genetic sex identification of experimental group samples using the competitive allele-specific PCR assays. Note: X-axis is FAM fluorescent unit, Y-axis is HEX fluorescent unit. The point near the Y-axis is homozygous, and the point in the middle is heterozygous. Green dots indicate male, red dots indicate female, blue dots indicate control of known sex, and black dots indicate control of water. Thirty-six muscle samples of 50 days and eighteen muscle samples of 80 days were analyzed in the experimental group (male:female 1:1).

The results of competitive allele-specific PCR genotyping for genetic sex identification are shown in Figure 8. A total of 54 individuals in the experimental group (36 samples from 50 days and 18 from samples of 80 days with a 1:1 sex ratio) were genotyped. The results of the genetic sex identification were consistent with the observations of histological analysis of the gonads and the shape of the abdominal flap.

### 3.6. The Expression of Pt-IAG, Pt-IGFBP and Pt-IR

**Figure 9:**
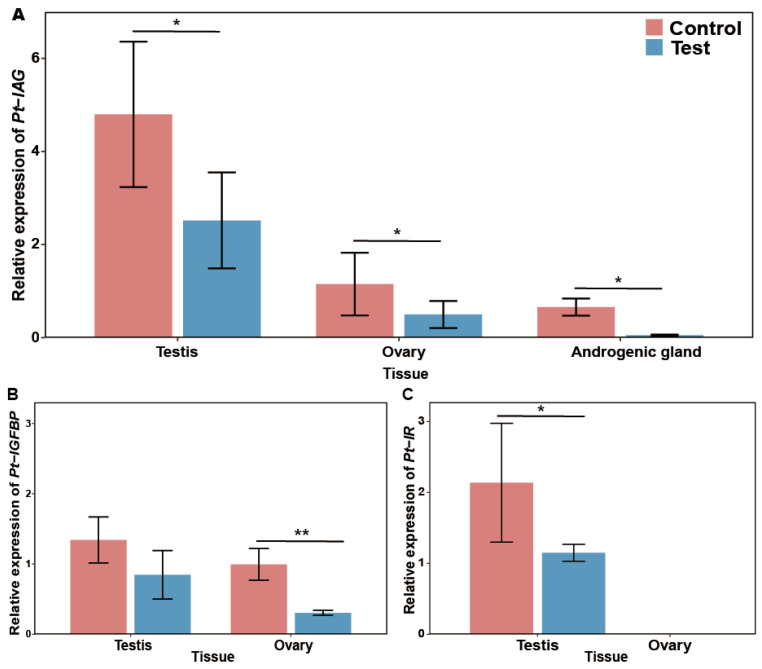
Relative expression of the *Pt-IAG* gene and related genes at the 50th day. Note: (**A**) The relative expression of *Pt-IAG* gene in testis, ovary and androgenic gland; (**B**) the relative expression of *Pt-IGFBP* gene in testis and ovary; (**C**) the relative expression of *Pt-IR* gene in testis and ovary. Data are presented as mean ± SD (*n* = 6, three technical replicates were performed for each sample) and were normalized to the control group. Significant differences between groups are indicated by asterisks (* for *p* < 0.05, ** for *p* < 0.01) based on independent-samples *t*-tests.

The mRNA expression of *Pt-IAG, Pt-IGFBP* and *Pt-IR* of the two groups at day 50 is presented in Figure 9. In the androgenic gland, the *Pt-IAG* gene was significantly higher in the control group (0.66 ± 0.18) than in the experimental group (0.05 ± 0.01), with a knockdown efficiency of 92.42% (*p* = 0.029). Similarly, in both the testis (control = 4.79 ± 1.56, experimental = 2.52 ± 1.03) and ovary (control = 1.15 ± 0.67, experimental = 0.50 ± 0.29), *Pt-IAG* expression was significantly higher in the control group (*p*_testis_ = 0.033, *p*_ovary_ = 0.019), with average knockdown efficiencies of 47.47% and 56.82%, respectively. *Pt-IGFBP* expression in the ovaries of the experimental group was significantly lower than that in the control group (control = 0.99 ± 0.22, experimental = 0.30 ± 0.03),representing a 69.7% reduction (*p* = 0.008). In contrast, testicular *Pt-IGFBP* levels showed no significant change between the two groups (control = 1.34 ± 0.33, experimental = 0.85 ± 0.35), with a 36.6% decrease that was not statistically significant (*p* = 0.146). *Pt-IR* expression in the testis of the experimental group was significantly lower than that in the control group (control = 2.14 ± 0.59, experimental = 1.15 ± 0.12), corresponding to a 46.3% reduction in the experimental group (*p* = 0.02). Notably, the *Pt-IR* transcript was not detected in the ovary under the present experimental conditions.

## 4. Discussion

The function of the *IAG* gene in crustacean sexual differentiation has been extensively documented in *M. rosenbergii* [3], *E. sinensis* [22], and *Penaeus vannamei* [53]. In the present study, we utilized siRNA knockdown to investigate the function of *Pt-IAG* in *P. trituberculatus*. The success of RNAi depends on precise siRNA design [54]. Our characterization of the *Pt-IAG* revealed a prototypical linear organization comprising a signal peptide, Bchain, Cpeptide, and A-chain, stabilized by inter-chain and intra-A-chain disulfide bonds. Notably, the highly effective siRNA identified here targeted a conserved domain within the A-chain, aligning with knockdown sites reported in previous studies [4,8]. Inthe mammalian insulin family, the A-chain serves as the primary effector for receptor binding [55], and similar in vitro recognition has been observed in *C. quadricarinatus* [12]. These structural similarities suggest that the A-chain may play an important role in receptor interaction, consistent with observations in other species.

The IAG hormone is traditionally recognized as the master regulator of sexual differentiation in crustaceans. Notably, *IAG* silencing in *M. rosenbergii* remains the only RNAi-based technology to date successfully developed to a commercial scale for aquaculture, underscoring its immense industrial potential [21,56]. However, in *P. trituberculatus*, *Pt-IAG* knockdown failed to trigger a phenotypic sex switch. This discrepancy suggests that the sex-determination hierarchy in *P. trituberculatus* possesses a higher degree of developmental robustness. We attribute this to several potential factors: (i) Insufficient continuity of interference pressure, as *Pt-IAG* transcription recovered 24h post-injection. (ii) Misalignment of the intervention window, as *Pt-IAG* is expressed as early as the zygote stage and secondary sexual characteristics emerge during the C I–C III stages [10]. The early expression of *Pt-IAG* suggests a potential role during early development, although its functional significance at these stages remains unclear. This may render the established male phenotype resistant to late-stage hormonal fluctuations. Additionally, (iii) sub-threshold knockdown efficiency and (iv) functional redundancy, where other *Pt-ILPs* may exert compensatory effects [57]. Collectively, these findings imply that *Pt-IAG* in swimming crabs may function as a component of a regulatory network rather than a binary “on/off” switch.

Beyond its primary synthesis in AG, the detection of *Pt-IAG* transcripts in the testis, ovary, and embryos (from zygote to heartbeat) challenges the classical “AG-exclusive” paradigm. This expression suggests a potential role during early development, although its functional significance at these stages remains unclear. This aligns with observations in *M. nipponense* [58] and *E. sinensis* [22]. Furthermore, the presence of these transcripts in gonads, reinforced by recent detection in female juvenile crab [10], implies a shift toward localized autocrine or paracrine regulation. Consequently, we propose a model of *Pt-IAG*that appears to contribute to growth regulation and reproductive development from the earliest life stages.

In vertebrates, insulin-like genes modulate spermatogenesis and oocyte development by mediating sex hormone signaling [59,60]. Our observation that *Pt-IAG* knockdown resulted in spermatogenic arrest and delayed ovarian maturation underscores its essential role in the gonadogenesis of *P. trituberculatus*. This functional conservation across species such as *Fenneropenaeus chinensis* [61] and *L. vittata* [62] highlights that its requirement for germline maturation is a conserved trait in decapods. Beyond reproduction, the significant reduction in body weight following knockdown—followed by compensatory gain—suggests that *Pt-IAG* appears to contribute to growth regulation. The consistency of this role across species suggests that *M. rosenbergii* [63], *M. nipponense* [64] and *L. vittata* [62] suggests that IAG signaling may be involved in integrating nutritional status with physical growth.

Based on the regulatory functions of *IGFBP* and *IR*, we monitored the expression of *Pt-IGFBP* and *Pt-IR*. Both genes were downregulated in males, aligning with the findings of Jiang et al. (2020) [8], who reported that short-term silencing of *Pt-IAG* lowers *Pt-IR* and *Pt-IGFBP* transcripts. Similar findings have been reported in other crustaceans, such as *M. rosenbergii* and *M. nipponense* [11,13]. Notably, the absence of detectable *Pt-IR* expression in ovaries suggests potential sex-specific differences in signaling pathways, although further validation is required. While similar tissue-specific patterns have been documented in *P. vannamei* [65] and *S. paramamosain* [66], the underlying mechanisms remain elusive. This potentially involves unidentified insulin-receptor isoforms or indirect modulation via metabolic tissues like the hepatopancreas. This divergence suggests that IAG signals are interpreted differently depending on the sexual and metabolic context of the target tissue.

Overall, these findings support a role for *Pt-IAG* in coordinating growth and gonadal development in *P. trituberculatus.* While this study underscores the pivotal role of *Pt-IAG*, certain limitations remain. Specifically, the lack of a non-targeting siRNA control and the reliance on a single reference gene (*RPL18*) for qRT-PCR normalization should be addressed in future work. Furthermore, although the effect sizes observed are substantial, they should be interpreted with caution given the small cohort size. Future research utilizing in vitro molecular tools—such as luciferase reporter assays and protein-interaction analysis—is essential to clarify the direct signaling mechanisms at the cellular level. Nevertheless, this study provides significant evidence for the multifaceted functions of *IAG* in crustaceans.

## 5. Conclusions

In summary, the *Pt-IAG* transcript was detected at the zygote stage, suggesting a potential role during early embryonic development before the initiation of morphological gonadal differentiation. Continuous knockdown of the *Pt-IAG* gene impaired the development of both male and female gonads. Furthermore, this treatment led to a significant decrease in body weight, underscoring the pleiotropic nature of *IAG* in coordinating both reproductive maturation and somatic growth. The absence of sex reversal may be related to limitations in RNAi duration and timing relative to critical developmental windows. These results suggest a possible association between *Pt-IAG* and insulin-like signaling pathways in males. In contrast, the distinct expression patterns in females may indicate sex-specific differences in receptor expression or signaling pathways. Collectively, our findings provide a preliminary conceptual framework for understanding the multifaceted and sex-dependent functions of IAG in decapod crustaceans.

## Figures and Tables

**Table 1:** Summary of *IAG* sequences used in phylogenetic analysis.

Sequence	Species	GenBank Accession No.
*Mr-IAG*	*M. rosenbergii*	AWU67706.1
*Sp-IAG*	*S. paramamosain*	AFY09905.1
*Es-IAG*	*E. sinensis*	AVK43106.1
*Cqui-IAG*	*Chaceon quinquedens*	ASA45642.1
*Cs-IAG*	*C. sapidus*	AEI72263.1
*Cs-IAG-ov*	*C. sapidus*	APC42865.1
*Mn-IAG*	*M. nipponense*	AHA33389.1
*Mv-IAG*	*Macrobrachium vollenhovenii*	AHZ34725.1
*Sv-IAG*	*S. verreauxi*	AHY99679.1
*Lvit-IAG1*	*Lysmata vittata*	QOD42428.1
*Lvit-IAG2*	*L. vittata*	QOD42429.1

**Table 2:** Primers used in this study.

Primer	Sequence (5′-3′)	PCR Objective	Reference
PS7FAM	GAAGGTGACCAAGTTCATGCTATTTTTG TACACTACACCTCCCCC	Competitive allele-specific PCR	[40]
PS7HEX	GAAGGTCGGAGTCAACGGATTTTTGTA CACTACACCTCCCCT	Competitive allele-specific PCR	[40]
PS7C	GCTAGAAAGGGRTGTA–AACAAGTT	Competitive allele-specific PCR	[40]
*Pt*-104-F	GCAGCUACUUCUUGUUUAUTT	RNAi	
*Pt*-104-R	AUAAACAAGAAGUAGCUGCTT	RNAi	
*Pt*-598-F	CUCCUAGCUUCCCGAAAUATT	RNAi	
*Pt*-598-R	UAUUUCGGGAAGCUAGGAGTT	RNAi	
*Pt*-301-F	CCUCAGCGAAAGACAUAAATT	RNAi	
*Pt*-301-R	UUUAUGUCUUUCGCUGAGGTT	RNAi	
*Pt*-744-F	CAGUAAAGUAGUAAUGUAATT	RNAi	
*Pt*-744-R	UUACAUUACUACUUUACUGTT	RNAi	
*Pt-IAG*-F	TAGTGGAACAAAACGAAGACC	qRT-PCR	
*Pt-IAG*-R	ATTTAGAGTAGCAGTAGACGC	qRT-PCR	
*Pt-IR*-F	AGAAGGTGCCCAGGAACTAAA	qRT-PCR	[8]
*Pt-IR*-R	AGGTGAGGTTGGATCGGAAT	qRT-PCR	[8]
*Pt-IGFBP*-F	TTACCACTATTGACGGCACCT	qRT-PCR	[8]
*Pt-IGFBP*-R	TCATTATC TGTACCCATCCTGTT	qRT-PCR	[8]
*RPL-18*-F	GCACTGTCACCGATGACCTC	qRT-PCR	[41]
*RPL-18*-R	CCTTGCACCAGCAGAGTGTT	qRT-PCR	[41]

## Data Availability

The original contributions presented in this study are included in the article and Supplementary Materials. Further inquiries can be directed to the corresponding author.

## Supplementary Materials

The following supporting information can be downloaded at: https://www.mdpi.com/article/10.3390/ani16091413/s1, Figure S1: Melting curve analysis of primer specificity; Table S1: Morphological description of embryonic and juvenile developmental stages in Portunus trituberculatus.; Table S2: Summary of qRT-PCR efficiency of the primer pairs; Table S3: (a) The Ct values (mean ± SD) of RPL_18 gene expressed in different tissue for adult crab; (b) The stability values of RPL_18 gene by BestKeeper analysis; Table S4: Macroscopic and histological criteria for ovarian developmental stages (I–V); Table S5: Macroscopic and histological criteria for testis developmental stages (I–III); Table S6: Effect sizes (Cohen’s d) for comparisons between the siRNA-PtIAG group and the control group.

## Abbreviations

The following abbreviations are used in this manuscript:

IAG	Insulin-like androgenic gland hormone
AG	Androgenic gland
RNAi	RNA interference
siRNA	Small interfering RNA
IGFBP	Insulin-like growth factor-binding protein
IR	Insulin receptor
ILP	Insulin-like peptide
dsRNA	Double-stranded RNA
Aa	Amino acid
BW	Body weight
CV	Coefficient of variation
qRT-PCR	Quantitative real-time PCR
