# Supplementary material for: Effects of RNA Interference-Mediated Silencing of the Insulin-Like Androgenic Gland Hormone Gene on Growth and Gonad Development in the Swimming Crab (Portunus trituberculatus)
Source: Animals (Basel). 2026 May 5;16(9):1413. doi: 10.3390/ani16091413 (PMC13162967; doi:10.3390/ani16091413)
Supplement: Supplementary file 1 [file animals-16-01413-s001.zip › Supplementary Tables.pdf]

Table S1.Morphological description of embryonic and juvenile developmental stages in *Portunus trituberculatus*.

Stage	Key Morphological Characteristics
Zygote Stage	Round or oval zygotes are densely packed on the outer side of the egg sheath, appearing dark in color and covered by a colorless, transparent vitelline membrane.
Cleavage Stage	The embryo surface is covered by a transparent vitelline membrane, with clear cleavage furrows visible.
Blastula Stage	Cleavage furrows disappear from the embryo surface, which becomes uniformly dense without thick structures; a mass of blastomeres is arranged on the surface, enclosed by a thin embryonic envelope.
Eye-pigment Stage	Optic lobe, antennular, and mandibular primordia form, with appendages reaching seven pairs; the yolk area decreases, the transparent area expands, and silvery eye-spot stripes appear on both sides.
Heartbeat Stage	The pigment area of the compound eyes expands and darkens; the cardiac primordium appears on the dorsal side of the yolk sac, and a low, irregular heartbeat can be observed.
Zoea I-IV Stages	The dorsum bears a distinct dorsal spine, and the telson furcae is long and prominent. Stages are distinguished based on the number of molts (four molts in total) and size differences observed under a microscope.
Megalopae Stage	After the fifth molt, metamorphosis takes place: chelipeds and large compound eyes appear, the dorsal spine disappears, and the telson furcae shortens.
Juvenile Crab I Stage	Following another molt from the megalopae stage, the telson furcae further shortens and degenerates, and the morphology closely resembles that of a subadult.

Table S2. Summary of qRT-PCR efficiency of the primer pairs.

Gene (Genbank Accession numbers)	Primer pairs	Annealing temperature (℃)	Amplification efficiency (%)
Pt-IAG(MH119940.1)	*Pt-IAG*-F/*Pt-IAG*-R	60℃	91%
Pt-IR(MT912035.1)	*Pt-IR*-F/*Pt-IR*-R	60℃	90%
Pt-IGFBP(MG735729)	*Pt-IGFBP*-F/*Pt-IGFBP*-R	60℃	93%
RPL-18(XM_045272895)	*RPL-18*-F/*RPL-18*-R	60℃	101%

Table S3-a. The Ct values (mean±SD) of *RPL_18* gene expressed in different tissue for adult crab.

Tissue	RPL_18
muscle	19.47±0.66
testis	18.90±0.86
androgenic gland	18.65±0.39
ovary	18.60±0.33

Table S3-b. The stability values of *RPL_18* gene by BestKeeper analysis.

Gene	n	Bestkeeper (SD value)
RPL18	12	0.49

Table S4. Macroscopic and histological criteria for ovarian developmental stages (I–V).

Stage	Macroscopic Characteristics	Histological Characteristics
Stage I	Translucent, smooth surface	Strongly basophilic cytoplasm; dominated by OG in early phase (mean length 9.70 ± 0.17 mm); large amounts of PRO and small amounts of EN present in late phase.
Stage II	Creamy white ovarian tissue visible	Strongly basophilic cytoplasm; dominated by EN (mean length 49.19 ± 0.68 mm); coexisting with PRO (mean length 28.47 ± 0.44 mm).
Stage III	Light yellow, increased tissue volume	Yolk granules appear; cytoplasm shifts from basophilic to eosinophilic; only a small number of irregular EN visible in the central region of ovarian lobules.
Stage IV	Orange lobes, expanded volume; covers ~50% of dorsal hepatopancreas	Cytoplasm remains eosinophilic; large amounts of yolk granules (YG) present in NO; mean length 245.32 ± 2.05 mm.
Stage V	Bright orange, occupies >50% of dorsal space within carapace	Yolk granules evenly distributed throughout cytoplasm; nucleus located peripherally and not easily discernible; mean length 324.88 ± 2.97 mm.

Table S5. Macroscopic and histological criteria for testis developmental stages (I–III).

Stage	Macroscopic Characteristics	Histological Characteristics
Stage I (Spermatogenesis)	Testis: Translucent or creamy white, band-shaped, small.Vas deferens: Barely visible before July, appearing as two parallel tubules in early August.	The testis is primarily composed of basophilic spermatogonia and primary spermatocytes, with increasing numbers of primary/secondary spermatocytes and a few spermatids by early August; only a small number of spermatophores are present in the vas deferens.
Stage II (Spermatophore Formation)	Testis: Creamy white, band-shaped, enlarged;Vas deferens: Clearly visible, differentiated into a flocculent anterior segment, a pink-white gelatinous middle segment, and a transparent coiled posterior segment.	The testis is dominated by secondary spermatocytes and spermatids, with occasional mature spermatozoa. The vas deferens contains loosely arranged triangular primary spermatophores in the anterior segment and numerous oval/round secondary spermatophores (~129.7 × 110.8 μm) in the middle segment, while the posterior segment contains only secretions. Spermatophores are basophilic, and secretions are eosinophilic in the anterior/middle segments with basophilic granules in the middle/posterior segments.
Stage III (Maturation)	Testis: Slightly reduced in size compared to Stage II;Vas deferens: Lumen significantly thickened and turgid.	The testis was dominated by strongly basophilic spermatids and spermatozoa. In the vas deferens, the primary (anterior) and secondary (middle, averaging ~273.9 × 257.2 μm) spermatophores were enlarged, increased in number, and densely

	packed, while the posterior segment contained no spermatophores but abundant flake-like eosinophilic and a small amount of granular basophilic secretions.

Table S6. Effect sizes (Cohen's d) for comparisons between the siRNA-*PtIAG* group and the control group.

Time Point	Parameter	Cohen's d
Day 50	Body Weight (male)	1
	Body Weight (female)	0.478
	Pt-IAG Expression(testis)	0.75
	Pt-IAG Expression(ovary)	0.465
	Pt-IAG Expression(androgenic gland)	1
	Pt-IGFBP Expression(testis)	0.615
	Pt-IGFBP Expression(ovary)	1
	Pt-IR Expression (testis)	0.903
Day 80	Body Weight (male)	1
	Body Weight (female)	0.086

Note: Cohen's d was interpreted based on the benchmarks of small (0.2), medium (0.5), and large (0.8) effect sizes.
