# Supplementary figures and images for: Effects of RNA Interference-Mediated Silencing of the Insulin-Like Androgenic Gland Hormone Gene on Growth and Gonad Development in the Swimming Crab (Portunus trituberculatus)
Source: Animals (Basel). 2026 May 5;16(9):1413. doi: 10.3390/ani16091413 (PMC13162967; doi:10.3390/ani16091413)

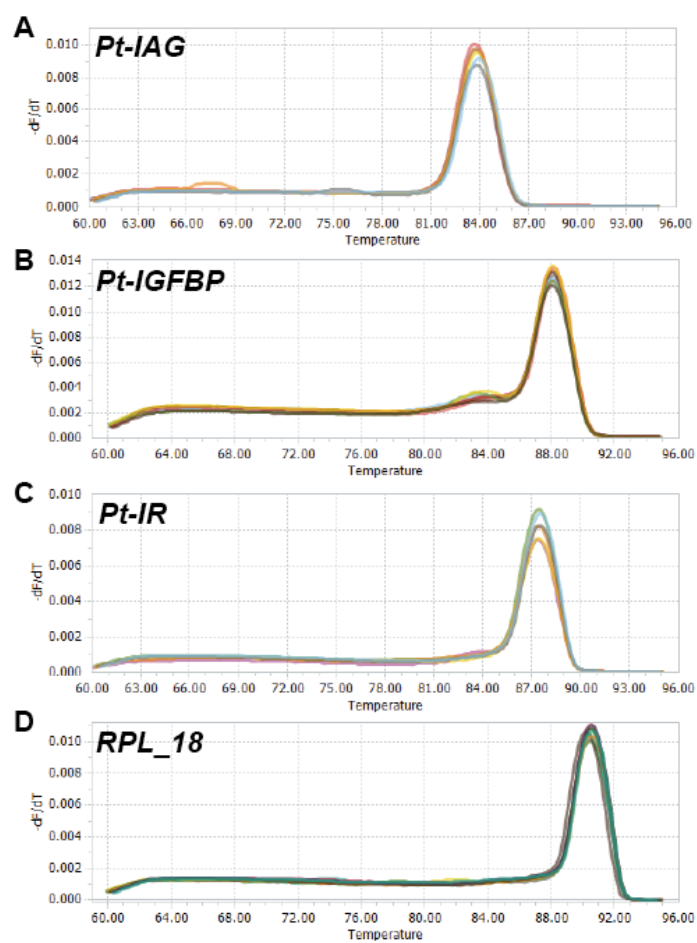

**Figure S1.** Melting curve analysis of primer specificity.

Supplement: Supplementary file 1 [file animals-16-01413-s001.zip › Supplementary Figures.pdf]
